# Changing the face of thyroid eye disease

**DOI:** 10.1038/s41433-022-02186-0

**Published:** 2022-07-26

**Authors:** Shoaib Ugradar, Robert A. Goldberg, Raymond S. Douglas

**Affiliations:** 1 The Jules Stein Eye Institute University of California, Los Angeles, CA USA; 2 Cedars-Sinai Medical Center, Los Angeles, CA USA

**Keywords:** Anatomy, Medical research

The history of medicine often shows us that a novel idea is not as novel as it may first appear. This is the case with thyroid associated orbitopathy (TAO). It was first described between 1040 and 1136 AD as a systemic disorder relating enlargement of the thyroid gland to exophthalmos [1]. This concept of a systemic condition causing exophthalmos was later readdressed in greater detail by Parry (1786) and Graves (1835). The condition being named after the latter [2].

Over the years, our understanding of Graves’ disease (GD) has increased. We now know that it is the most common cause of hyperthyroidism and is responsible for a myriad of signs including; a goitre, a resting tremor, tachycardia, hyperreflexia, lid lag, skin changes and proximal myopathy [3]. Thyroid dermopathy (pretibial myxoedema), a nodular or diffuse thickening of the pretibial skin is found in 13% of patients with GD [4]. Further, 20% of patients with dermopathy present with clubbing of the fingers and toes (thyroid acropachy) [5]. The most common extrathyroidal manifestation of GD is TAO, presenting in up to 50% of patients [6]. It is characterized by proptosis, chemosis, diplopia and in severe cases, loss of vision [5].

Anti–thyrotropin-receptor antibodies are common to virtually all patients with TAO and GD, suggesting that immunoreactivity against the thyrotropin receptor leads to the development of both [7]. Given the widespread signs of GD across the body, it is therefore plausible that the manifestations of TAO might also be associated with extra orbital changes.

Over the past three decades, several studies have focused on periorbital changes. In 1994, Hurwitz and colleagues reviewed orbital MRI from 14 patients (28 orbits) with TAO. They also had two control groups, the first consisting of 22 orbits from 13 patients without orbital pathology and the second with 12 orbits from 12 patients with proptosis caused by pathology other than GD [8]. Using a ruler to measure the cross section of the brow fat pad on axial scans, they found that TAO was associated with an increase in brow fat volume. This was exclusive to GD, since other patients with proptosis unrelated to GD did not have similar findings.

With advances in technology, Goldberg and colleagues used the validated Mimics (Version 9, Materialise, Leuven, Belgium) software [9] to quantify the volume of the lateral brow fat in patients with TAO and controls. Using bony landmarks, the superolateral brow was defined from the supraorbital notch to the lateral orbital rim. The inferior limit was defined as the zygomaticofrontal suture, while the superior extension was defined by a horizontal line across the brow, 0.5 cm from the level of the supraorbital notch. From 100 scans (48 with TAO and 52 controls), a significant increase in the lateral brow fat of patients with TAO was seen [10]. This study was limited by potential differences in body mass index between the TAO and control groups.

The same group also studied the histopathology of the brow fat in patients with TAO. They found an increase in expression of the insulin-like growth factor 1 receptor β (IGF-1Rβ) and the thyroid-stimulating hormone receptor (TSHR), in orbital and brow fat, compared to controls [11]. These studies led to the concept of a thyroid periorbitopathy.

Perry and colleagues further advanced this concept by reviewing volumetric changes in the soft tissue of the temporal fossa. In an age matched CT based case control study of 56 patients (28 with TAO and 28 controls), those with TAO had significantly increased fat and soft tissue volume in the temporal fossa compared to controls [12].

Changes in the brow appear to be noticeable by observers. In a retrospective cohort study of 75 patients (150 hemifaces) with confirmed TAO, a 4-point grading system was used to assess enlargement of brow fat on photographs using 6 masked observers. The average grade was 0.3 for premorbid eyes and 1.1 for morbid eyes. The authors found that tissue expansion in the brow region was associated with disfiguring features that included a change in shape of the eyebrow (lateral flare or “C” shape) [13]. Other changes that may be observable include malar / cheek swelling, described in 6 patients with TAO [14].

Teprotumumab, a monoclonal IGF-1R inhibitor, was recently approved for the specific treatment of TAO by the FDA. Recent studies have demonstrated an over expression of the IGF-1R on orbital fibroblasts (OFs) of patients with TAO and GD [15, 16]. Activation of the IGF-1R and the TSHR-signalling complex, stimulates OFs to release inflammatory cytokines and increase hyaluronan production [17]. These events contribute significantly to the expansion of soft tissue (fat and muscle). Therefore, teprotumumab reduces soft tissue expansion in TAO [18].

We also found that the effects of teprotumumab seem to be confined to tissue that has undergone expansion due to TAO. This was seen in a study of patients with asymmetric TAO. Following treatment, we found a significant change in proptosis and soft tissue volume in the affected orbit, without any significant change in the unaffected orbit [19].

**Fig. 1:**
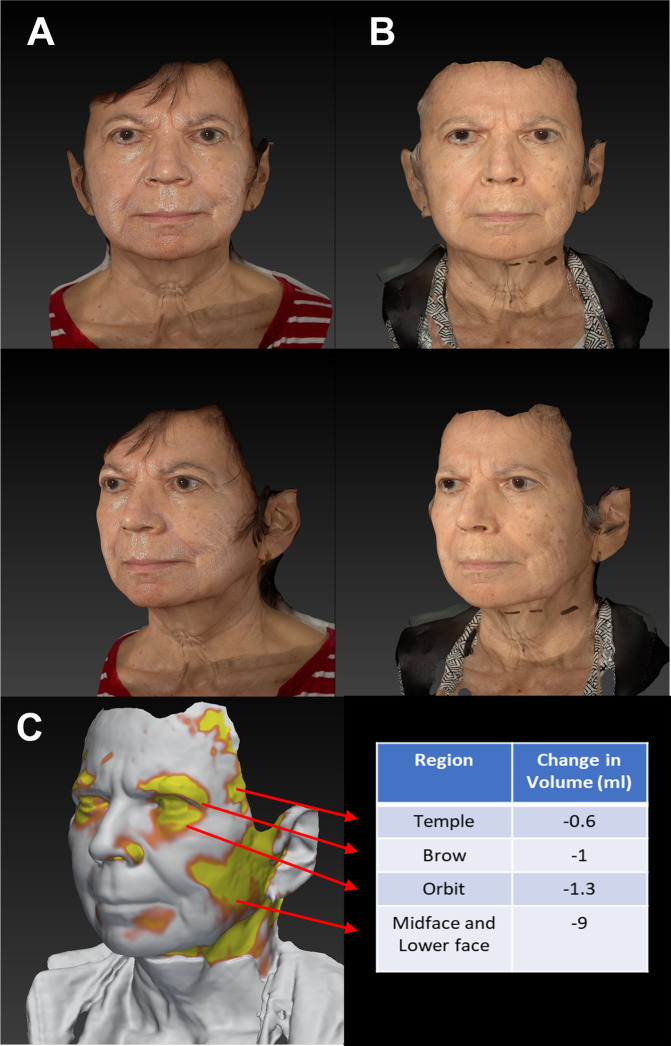
Facial changes following treatment of Thyroid eye disease. 3D stereophotogrammetric imaging pre (**A**) and post (**B**) Teprotumumab therapy. **C** Regional facial volumetric changes following treatment with Teprotumumab. The patient has not had any other interventions. The regions in yellow show the greatest decrease in volume following therapy. Permission has been obtained from the patient for publication of Fig. 1.

This led us to hypothesize that teprotumumab might also have an impact on TAO related soft tissue expansion on other regions of the face. In a prospective study of 23 patients with TAO undergoing treatment with teprotumumab, we performed 3D stereophotogrammetric imaging. Imaging was obtained at baseline and following completion of therapy. All patients were euthyroid at baseline and the mean (SD) duration of the disease was 29 (38) months. After treatment, there was a mean (SD) decrease of 8.9 mL (8.7) across the whole face. When the face was divided into regions, mean (SD) decrease in volume for each region was: 0.75 mL (0.84) in the upper face, 1.8 mL (1.3) in the periorbital region, 0.17 mL (0.5) in the temples, 1.62 mL (3.16) in the midface, and 2.67 mL (4.6) in the lower face. These changes were unrelated to changes in body weight or a history of steroid use (Fig. 1) [20].

In recent times, our understanding of TAO has evolved from the dogma of confining its range of influence to the orbits, to including the periorbital region and in the latest study, the entire face. The culmination of this work suggests that what was true in 1726 viz. that the exophthalmos (and therefore TAO) was part of a more widespread condition, is true now.

Unfortunately, given the significant burden of morbidity in TAO, it has a marked impact on quality of life [21] and mental health [22], leading to increased rates of suicide [23]. The psychological burden of the progressive disfigurement resulting from TAO is well established [24, 25]. In one study, changes in appearance were more disabling than changes in visual function [21].

These studies bring into sharp focus the importance of understanding that TAO not only involves the orbits but the entire face. This has implications for educating and counselling patients. Further, therapeutic measures aimed at treating TAO should not focus only on the orbital changes. This concept has obvious ramifications for surgical rehabilitation. Traditionally, this has focused on decompression of the orbit and correcting eyelid malposition. However, a pan facial approach may be more appropriate (Fig. 1)

While teprotumumab is currently the only FDA approved medication for TAO, there is a rapidly growing industry focusing on developing agents aimed at blocking the IGF-1R pathway. The introduction of such agents that could address the wide-reaching effects of TAO, may redefine the treatment landscape.
